# Study of thyroid function in pregnancy, its feto-maternal outcome; a prospective observational study

**DOI:** 10.1186/s12884-020-03448-z

**Published:** 2020-12-10

**Authors:** Kalpana Mahadik, Payal Choudhary, P. K. Roy

**Affiliations:** Department of Obstetrics & Gynecology, R.D. Gardi Medical College, Ujjain, Pin-456006 India

**Keywords:** Pregnancy and hypothyroidism, Subclinical hypothyroidism, Hypothyroidism and anemia, Thyroid dysfunction in pregnancy

## Abstract

**Background:**

Pregnancy is a stress test of maternal thyroid function. The prevalence of thyroid dysfunction in pregnant women is high. Subclinical hypothyroidism occurs in 10% of all pregnancies. Effects of hypothyroidism in pregnancy are anemia, low birth weight and mental retardation in neonate. This study is aimed to evaluate maternal and fetal outcomes in pregnant women with deranged thyroid profile. The relevance of this study is to document the association of hypothyroidism and its adverse effects on mother and fetus.

**Methods:**

This prospective observational study was carried out at R.D. Gardi Medical College, Ujjain, India. Subjects of this study were 198 antenatal women in third trimester with singleton pregnancy admitted in the obstetric ward, and informed consent was obtained. Women were chosen irrespective of age, parity, residence and socioeconomic status. Women with multiple pregnancy, a known case of thyroid disorder, or any pre-existing medical disorder were excluded. Routine hematological parameters and estimation of T3, T4 and TSH was conducted. Patients with deranged thyroid profile were subsequently assessed for maternal and fetal complications. History of infertility, family history of thyroid disease, menstrual pattern, recurrent abortion, hemoglobin level and fetal outcome were the main study variables. Data was analysed in SPSS software for statistical co-relation.

**Results:**

Prevalence of thyroid disorder is 11%; with subclinical hypothyroidism, overt hypothyroidism and subclinical hyperthyroidism occurring in 5.6, 3.5 and 1.5% of subjects respectively. In women with subclinical and overt hypothyroidism, anemia was present in 26.3% being significantly associated with hypothyroidism (*p* = 0.008). With respect to fetal outcome, LBW 31.6% (*p* = 0.001), NICU admission 42.1%, (*p* = 0.000) and low APGAR Score (21.1%, *p* = 0.042) were statistically associated with hypothyroidism. Risk of anemia, Low Birth weight, NICU admissions, and low APGAR score was 4.8, 6.3, 0.14 and 3.64 times higher respectively in women with hypothyroidism than in women who are euthyroid.

**Conclusion:**

Prevalence of subclinical hypothyroidism is 5.6% in 3rd trimester of pregnancy. Anemia, pre-eclampsia, high caesarean rates and neonatal morbidities is significantly associated with hypothyroidism.

## Background

Stress of pregnancy may result in clinical or sub clinical hypothyroidism in women with limited reserve. Reference ranges of TSH or free thyroxine (fT4) obtained from non-pregnant populations change in pregnant women because of the physiological changes in thyroid function in pregnancy. Physiological and hormonal changes in pregnancy result in increased production of thyroxin (T4) and triiodothyronine (T3) by up to 50%, leading to an increase in a woman’s daily iodide requirement, while thyroid-stimulating hormone (TSH) levels decrease, especially in the first trimester [1]. As Human Chorionic Gonadotrophin (HCG) is thyrotrophic its high levels specially in 1st trimester result in low TSH values and thus cut offs become less. In women with low thyroid reserves stress of pregnancy manifests as overt disease [2]. In an iodide sufficient area, thyroid adaptations are well tolerated, as stored inner thyroid iodide is adequate; however, in iodide deficient areas, these physiological adaptations lead to significant changes in pregnancy [3]. Hypothyroidism is widely prevalent in pregnant women and the rate of detection, especially in a developing country like India, has not kept pace with the magnitude of the problem. Since hypothyroidism is easily treated, timely detection and treatment of the dysfunction could reduce the burden of adverse fetal and maternal outcomes in pregnancy which are commonly encountered. Prevalence of overt thyroid dysfunction is 2–3% in pregnant women, subclinical dysfunction is 10%, while rate of autoimmunity is 5–10% [4, 5].

Maternal complications include miscarriage, anemia, preeclampsia, gestational hypertension, placental abruption, preterm delivery, increased rate of caesarean section, and postpartum hemorrhage. The mode of delivery may have adverse impacts on fetal-pituitary-thyroid axis. Fetal outcomes resulting from thyroid dysfunction are preterm birth, neonatal respiratory distress syndrome, low birth weight (LBW), perinatal morbidity and mortality, increased NICU admission; and neuropsychological and cognitive impairment. Thyroid hormone is critical for brain development in the developing fetus. Children born with congenital hypothyroidism have severe cognitive, neurological and development abnormalities if the condition is not recognized and treated promptly. A study demonstrated that children born to pregnant women with hypothyroidism had lower intelligence quotient (IQ) scores compared to children born to pregnant women without hypothyroidism [6].

For these reasons, it is important to adopt appropriate strategies to identify women at risk of these adverse outcomes and to implement screening tools for early detection and initiation of effective treatment. Controversies in trimester specific reference range in various demographic sites and number of reports being few in developing countries relevance of the study is high. In light of the high prevalence of thyroid disturbances in pregnancy and associated risk to mother and fetus, this study aims to determine the prevalence of thyroid disorders in pregnancy and its maternal and fetal outcomes in a tertiary care facility in Central India.

## Methods

This observational study was carried out at R. D. Gardi Medical College, Ujjain, India. We recruited 198 antenatal women in third trimester admitted into the obstetric ward with singleton pregnancy for other obstetric indications. Informed consent was obtained from all subjects. Subjects were chosen irrespective of age, parity, residence and socioeconomic status. Women with multiple pregnancies, a known case of thyroid disorder, on any treatment or with any pre-existing medical disorder, such as diabetes mellitus, or cardiac or pulmonary disease were excluded. Routine hematological parameters and estimation of T3, T4 and TSH was conducted. Patients with a deranged thyroid profile were subsequently assessed for maternal and fetal complications. Infertility, family history of thyroid disorder, menstrual history, recurrent abortions, mean T3, T4, TSH levels, haemoglobin levels, maternal and fetal outcome were the main study variables. Uni variate analysis was conducted to assess co-relation of thyroid disorders with other clinical features like menstrual rhythm, infertility, family history of thyroid disorder and miscarriage.

Estimation for TSH was conducted using the Enhanced Chemiluminescence method. Estimation of free T3 and free T4 was subsequently carried out when TSH levels were abnormal. Cut off values used for TSH were those indicated by the American Pregnancy and Thyroid Association: 1st trimester: 0.1–4.0mIU/L, 2nd trimester: 0.2–4.5mIU/L, 3rd trimester: 0.3 -5mIU/L [7]. Normal free T4 level is 0.7 to1.8 ng/dl and free T3 level is 1.7 to 4.2 pg/ml. Patients with normal fT4 and high TSH were considered to have subclinical hypothyroidism (SCH); those with low fT4 and high TSH were considered to have overt hypothyroidism; those with normalfT4 and low TSH were considered to have subclinical hyperthyroidism; and those with highT4 and low TSH were considered to have overt hyperthyroidism [7].

Maternal co-morbidities pertaining to thyroid dysfunction include history of miscarriage, anemia (haemoglobin level less than 10 g/dl), preeclampsia (blood pressure more than 140/90 with proteinuria after 20 weeks gestation), gestational hypertension (blood pressure more than 140/90 without proteinuria after 20 weeks gestation), oligohydramnios (amniotic fluid Index ≤5), preterm delivery (delivery before completion of 37 weeks of gestation) and increased rate of caesarean section. Fetal outcomes include LBW (neonatal birth weight less than 2.5 kg), Low Apgar score (1-min Apgar less than 5), and increased NICU admission.

Data management and analysis was conducted using Statistical Package for the Social Sciences (SPSS Version 25). The categorical variables were assessed using Pearson chi-square test. Association of risk factors was calculated by binary logistic regression. The test was considered significant only when the *p* value is less than 0.05. The study protocol was approved by the Scientific and Ethical Committee of the Institution. All the participants were also informed about the study procedure and the information that was required from them for the study. Written consent was obtained.

## Results

Of the198 women screened, 22 (11%) had abnormal thyroid function. Prevalence of subclinical hypothyroidism, overt hypothyroidism, and subclinical hyperthyroidism was 5.6% (*n* = 11), 3.5% (*n* = 7), and 1.5% (*n* = 3), respectively demonstrating that the occurrence of subclinical hypothyroidism is more common during pregnancy. One sample was showing high TSH and slightly above normal T4. For purpose of risk factors, it was included in hypothyroidism, thereby denominator for risk factors is 19.

Mean serum TSH levels among women with subclinical hypothyroidism, overt hypothyroidism and subclinical hyperthyroidism were 8.02 ± 1.25mIU/ml, 11.92 ± 5.34mIU/ml and 0.07 ± 0.03mIU/ml, respectively. Mean serum fT3 levels among women with subclinical hypothyroidism, overt hypothyroidism and subclinical hyperthyroidism were 2.92 ± 0.454 pg/ml, 1.58 ± 1.43 pg/ml, and 4.16 ± 0.40 pg/ml, respectively. Mean serum fT4 levels among women with subclinical hypothyroidism, overt hypothyroidism and subclinical hyperthyroidism were 1.09 ± 0.30 ng/dl, 0.36 ± 0.24 ng/dl and 1.2 ± 0.10 ng/dl, respectively. (Table 1).

**Table 1:** Prevalence of thyroid disorders in 3rd trimester of pregnancy

Thyroid status	Prevalence	Mean TSH (mIU/L)	Mean fT4 (ng/dl)	Mean fT3 (pg/ml)
Subclinical hypothyroidism *n* = 11	5.6%	8.02 ± 1.25	1.09 ± 0.30	3.07 ± 0.56
Overt hypothyroidism *n* = 7	3.5%	11.92 ± 5.34	0.36 ± 0.24	0.81 ± 0.66
Subclinical hyperthyroidism *n* = 3	1.5%	0.07 ± 0.03	1.2 ± 0.10	4.1 ± 0.40

Of the 22 women with dysfunction, 22.7% had a history of irregular menstrual rhythm; 4.5% had history of infertility treatment; 4.5% had family history of thyroid disorder and 4.5% had history of recurrent miscarriage. There was no statistically significant association between any of these factors and the occurrence of thyroid disorder (*p* values were 0.655, 0.217, 0.079, and 0.752, respectively) (Table 2).

**Table 2:** Prevalence and associated risk factors in thyroid disorder

Risk factors	%(n)	*P* value
Irregular menstrual rhythm	22.7% (5)	0.655
History of infertility treatment	4.5% (1)	0.217
Family history of thyroid disorder	4.5% (1)	0.079
Miscarriage	4.5% (1)	0.752

Of the women with hypothyroidism, 26.3% had anaemia, and the association between occurrence of hypothyroidism and anaemia was statistically significant (*p* = 0.008). Preeclampsia was observed in 15.8% of women, and the association between occurrence of hypothyroidism and preeclampsia was statistically significant(*p* = 0.041). Cesarean delivery occurred in 26.3% of women with hypothyroidism having significant association (*p* = 0.012) and oligohydramnios (*p* = 0.072). Preterm delivery occurred in 5.3% of hypothyroidism and was not significantly associated with hypothyroidism. 31.6% had LBW babies, and the association between LBW and hypothyroidism was significant (*p* = 0.001). For 1-min Apgar score cut off value considered was 5, as an indicator for fetal asphyxia. Out of 19 hypothyroid women 4 (21.1%) had babies with low Apgar scores which was significantly associated (*p* = 0.042). NICU admission 42.1% was significantly associated with hypothyroidism (*p* = 0.000).

The risk of anemia in women with hypothyroidism is 4.8 times (95% CI = 1.5–15.8) higher than in women with euthyroidism. It is likely that hypothyroidism may add to the severity of anaemia.

Risk of delivery of LBW babies is 6.3 times higher in women with hypothyroidism (95% CI = 2.03–19.5) than in women with euthyroidism. Risk of NICU admission and low Apgar score were 0.14 times (95% CI = 0.048–0.39) and 3.6 times (95% CI = 1.04–12.7) higher in babies born to women with hypothyroidism compared to those born to women with euthyroidism (Table 3).

**Table 3:** Association of maternal and fetal risk factors in women with hypothyroidism (*n* = 19)

Outcome %(n)	95% CI	Odds Ratio	*p* value
Anaemia 26.3% (5)	1.50–15.8	4.88	0.008
Preeclampsia 15.8% (3)	1.06–19.22	4.52	0.041
Preterm 5.3% (1)	0.253–22.54	2.39	0.447
Oligohydramnios 10.5% (2)	0.034–1.15	0.19	0.072
Caesarean section 26.3% (5)	1.39–14.38	4.47	0.012
Low birth weight (LBW) 31.6% (6)	2.03–19.54	6.30	0.001
Low Apgar Score 21.1% (4)	1.04–12.70	3.64	0.042
NICU admission 42.1% (8)	0.048–0.391	0.14	0.000

## Discussion

Thyroid disorder is common in women of reproductive age, and it is the most common endocrine disorders after diabetes in this age. Prevalence of thyroid disorders in pregnancy and its maternal and fetal complications in pregnant women vary greatly in different regions depending upon many factors. To date, there are few studies of thyroid function and pregnancy in Central India. The geographic location may be a factor in prevalence of thyroid disorder, because the amount of iodine in common salt and consumption of salt may vary from region to region. This study addresses issues related to abnormal thyroid function and feto-maternal outcomes in this setting.

### Prevalence of thyroid disorder

The laboratory technique Enhanced Chemiluminiscence used in the study is “Vitros ECi” an orthoclinical diagnostics which is FDA approved. First trimester values are more important than 2nd and 3rd trimester. Because it has an immense role in preventing maternal complications later on, if SCH is diagnosed early, treatment with low doses of levo-thyroxin will prevent it. Debate on upper limit for defining 1st trimester reference value is addressed in the Guideline [7]. The Task Force advises; definition for reference range be as per results for that particular population and laboratory technique of that Institute. If internal or transferable pregnancy-specific TSH reference ranges are not available, an upper reference limit of ∼4.0 mU/L may be used. For most assays, this limit addresses a reduction in non-pregnant TSH upper reference limit of ∼0.5 mU/L.

As this study was conducted in 3rd trimester pregnant women, we do not report any outcomes for 1st or late 1st trimester.

As per older guideline, considering TSH cut off values for each trimester as, 1st trimester: 0.1–2.5mIU/L, 2nd trimester: 0.2–3.0mIU/L, 3rd trimester: 0.3 -3mIU/L is questionable [2]. Only modest reductions in upper limit of TSH in 1st trimester from a non-pregnant value was documented by studies published from India and Korea after 2011. Limited availability of trimester specific reference ranges is an important factor in dis-similarity in values in different geographical and demographic sites. The concept of 1st trimester value of 2.5 mU/L as cut off for SCH and hypothyroidism is contextual in studies from India and China published in 2011 [8] and 2014 [9]. In view of scarcity of recent reports from India we follow standard ATA 2017 guideline.

The observed prevalence of thyroid disorder in 3rd trimester of pregnancy in the present study is 11%, which is comparable to the prevalence observed in a study conducted by Weiwei Wang et al. (10.2%) [8] and Ajmani et al. (13.25%) [9]. Variations in different areas may be due to non-uniformity in the study setting or in laboratory techniques, personal human error, and differences in sample size. In India, the prevalence of hypothyroidism in pregnancy is much higher compared to that in Western countries. Iodine deficiency could be a contributing cause. The percentage of households consuming iodised salt in India, as per the Iodine Network Global score card 2010, is 51% [10]. Hashimoto’s thyroiditis is a cause of hypothyroidism in iodine-sufficient areas, such as North America and Western Europe.

In the present study, the prevalence of subclinical hypothyroidism, overt hypothyroidism, and subclinical hyperthyroidism in pregnancy is 5.6, 3.5, and 1.5%, respectively (Table 1). This is in agreement with the findings of some Indian studies in which the prevalence of subclinical hypothyroidism and overt hypothyroidism is 6.1 and 0.7% respectively [11]. Another Indian study in 2016 reports prevalence of SCH 8% in 3rd trimester [12]. In a recent review and meta-analysis, prevalence rates reported were 0.50, 3.47, and 2.05% for overt hypothyroidism, subclinical hypothyroidism and isolated hypothyroxinaemia respectively [6].

We report mean serum TSH levels in women with subclinical hypothyroidism, overt hypothyroidism, and subclinical hyperthyroidism being 8.02 ± 1.25mIU/ml, 11.92 ± 5.34mIU/ml, and 0.07 ± 0.03mIU/ml respectively. Mean serum fT3 levels among women with subclinical hypothyroidism, overt hypothyroidism, and subclinical hyperthyroidism were 2.92 ± 0.454 pg/ml.,1.58 ± 1.43 pg/ml and 4.1 ± 0.40 pg/ml respectively. Mean serum fT4 levels among subclinical hypothyroid, overt hypothyroidism, and subclinical hyperthyroid women were 1.09 ± 0.30 ng/dl, 0.36 ± 0.24 ng/dl and 1.2 ± 0.10 ng/dl, respectively while a report from India in 2016 quotes reference values for TSH, fT3 and fT4 as 0.47–5.78 (uIU/ml), 0.24–3.61(ng/100 ml) and 0.47–5.1 (ng/100 ml) in 3rd trimester [12].

A recent review suggests that stricter criteria of TSH values with a 2.5 cut-off may be considered too low. Many women would be unnecessarily diagnosed as having SCH and may be subjected to the therapeutic burden of LT4 treatment [13].

### Risk factors

Thyroid dysfunction results in anovulatory cycles, luteal phase defect, high prolactin (PRL) levels, and sex hormone imbalances. All of these factors may result in infertility and irregular menstrual cycles, as documented by various authors [14]. In the present study, among women with hypothyroidism, 4.5% had a history of infertility treatment, compared to 3.8, and 4.0% women with hypothyroidism observed in other studies [15, 16]. We observed that, 22.7% of women with hypothyroidism had irregular menstrual rhythm.

Thyroid peroxidase (TPO) enzyme is responsible for the oxidation and organization of iodine, and for the formation of fT4 and fT3 hormones [17]. Thyroglobulin (TG) is a glycoprotein that acts as a substrate for synthesis and storage of thyroid hormones [18]. Autoimmune thyroid disorders present with antibodies to both resulting in hypothyroidism. Thyroid autoimmunity is associated with recurrent miscarriage likely to be due to generalized activation of the immune system and transplacental transfer of antibodies, causing fetal rejection [19, 20]. The presence of antibodies to thyroid peroxidase (TPO-Ab) or thyroglobulin in pregnancy is associated with significant increase in miscarriages, premature deliveries, gestational diabetes, postpartum thyroiditis and permanent hypothyroidism [21–23]. In the present study, miscarriage rate in women with hypothyroidism was 4.2%, which is similar to results of other studies, reporting rates of 5.6 and 5.0% [8, 15]. Hypothyroidism in pregnancy has immense relevance in clinical obstetric abnormalities.

First-degree relatives of patients with hypothyroidism due to Hashimoto’s thyroiditis have a nine-fold higher risk of developing this disease compared to the general population [24]. Family history of thyroid disorder was seen in 4.5% of women with hypothyroidism, which is comparable to the prevalence observed in other studies: 12.7% [8].

In this study, no statistically significant association was observed between thyroid dysfunction and clinical obstetrics and gynecological features, including miscarriage, menstrual irregularity, family history of thyroid disorder, and infertility (Table 2).

### Association of Maternal and fetal outcome with thyroid disorder

#### Anemia

Iron deficiency causes impairment of the heme-dependent enzyme thyroid peroxidase, thereby limiting synthesis of thyroid hormones, which can lead to a reduction in circulating levels of tT3 and tT4. Iron repletion may reverse hypothyroidism [25]. In the present study, anemia was observed in 26.3% of women with hypothyroidism (*p* = 0.008) while other authors have observed occurrence of anemia in 4.2% of women with hypothyroidism [26]. In one study, prevalence of anemia in women with hypothyroidism was as high as 60% due to iron deficiency [27]. As per a report from North India anemia and hypothyroidism are very commonly associated [28]. It is likely that hypothyroidism may add to the severity of anemia. The results of this study support an important clinical picture of an association between anemia and hypothyroidism.

#### Pre-eclampsia

Hypothyroidism causes vascular smooth muscle contraction both in systemic and renal vessels, which leads to increased diastolic pressure, peripheral vascular resistance, and decreased tissue perfusion, which could be the pathophysiology of preeclampsia in hypothyroidism [29, 30]. Thyroid dysfunction can be associated with proteinuria, which is known to result in increased excretion of thyroxine and thyroid-binding globulins. Rare cases have been reported in which proteinuria is severe enough to result in losses of thyroid-binding globulins and thyroxine that cannot be compensated by the body [31–33]. In the present study, pre-eclampsia was observed in 15.8% of women (*p* = 0.041) with hypothyroidism. These results are comparable to those of other studies, in which preeclampsia was observed in 13.6% women with SCH and 14.7 in overt hypothyroidism [15, 34].

#### Cesarean section

Increased rate of cesarean delivery is another outcome, observed in 26.7% (*p* = 0.012) of women with hypothyroidism. Other authors have reported rates of cesarean delivery of 22.9% in women with hypothyroidism [34]. The reason for the increased risk of cesarean delivery may be due to the associated pregnancy complications, such as hypertensive disorders, gestational diabetes, and preterm birth. Whether otherwise uncomplicated hypothyroidism increases risk of cesarean section warrants further study [35–37]. Some authors reported, pre-eclampsia (*p* = < 0.001), preterm labor (*p* = 0.001) and abruption (*p* = 0.03) being significantly related to hypothyroidism [38].

Complications that were observed to have lower prevalence in women with hypothyroidism in this study were oligohydramnios (10.5%) and preterm labor (7.8%). These findings are similar to those of other reports [20, 27].

#### Fetal outcomes

Low birth weight is associated with hypothyroidism due to its association with preeclampsia. Reduced fetal thyroxine may cause disruption to the development of the pituitary-thyroid axis of the newborn, fetal pituitary growth hormone secretion, vascular responsiveness and maturation, and cardiovascular homeostasis in utero [39–41]. These factors are causative for the observation of reduced neonatal birth weight of offspring born to mothers with inadequately controlled hypothyroidism at initial presentation or at third trimester. In this study LBW was observed in 31.6% of women with hypothyroidism, as compared to 20% observed in another study [42].

NICU admission in thyroid dysfunction was 42.1%, which is similar to the rates of 46.6 and 42% [10, 42]. Low Apgar scores occurred in 21.1% of babies born to women with hypothyroidism, compared to 20% observed in another study [15].

We did not find Intrauterine death as a fetal complication of hypothyroidism, unlike the findings of one report [38]. In the present study, hypothyroidism was found to be significantly associated with LBW(*p* = 0.001) and NICU admission (*p* = 0.000) similar to study conducted by Gupta HP et al. [38].

Considering the results, we feel that estimation and diagnosis of thyroid parameters has high clinical relevance. However, there is an ongoing debate regarding cost-effectiveness of universal vs. targeted screening in pregnant women. Current recommendations suggest targeted TSH screening for women at high risk for thyroid disease before or during early pregnancy [7]. Recommendations also focus on TPOabs- positive and negative women. It states that risk of pregnancy loss is more in TPOabs- positives at 1st trimester cut off TSH 2.5 mU/L and more. In an RCT authors advice benefit of levothyroxine treatment around 9 weeks gestation [43]. They also document improvement in adverse pregnancy outcomes only in TPOabs-positive women with mild hypothyroidism (defined as a TSH > 2.5 mU/L) with thyroxin therapy. The Task Force advocates evaluation of TPOabs for asymptomatic women with higher TSH (2.5 mU/L) in first trimester. In this study we have not carried out TPOabs status of study subjects.

Based on our sample size (*n* = 198) the study is 80.7% powered for 8 independent variable comparison for the outcome, which is adequate.

### Implications

Reporting observed values of TSH, T4 and T3 in 3rd trimester of pregnancy will add to available literature. High association of hypothyroidism and various feto-maternal adverse outcomes again supports argument for universal screening for thyroid function in pregnancy.

### Limitations

Due to small sample size and variability in TSH estimation technique, we cannot authoritatively submit the values of TSH, T4, T3 in 3rd trimester of pregnancy.

## Conclusion

This study concludes that there is a high prevalence of thyroid dysfunction in pregnancy (11%) in Central India, with the majority of women being subclinical hypothyroidism. Association of maternal anemia, preeclampsia, increased cesarean delivery, presence of LBW babies, low Apgar score and increased number of NICU admission; is a major finding of this study.

## Data Availability

The datasets used and analyzed during the current study are available from the corresponding author on reasonable request.

## Abbreviations

TSH: Thyroid Stimulating Hormone
hCG: Human Chorionic Gonadotrophin
T4: Thyroxine
T3: Triiodothyronine
NICU: Neonatal Intensive Care Unit
fT4: Free Thyroxine
TRH: Thyroxine Releasing Hormone
TBG: Thyroxine Binding Globulin
TG: Thyroglobulin
IQ: Intelligence Quotient
tT3: Total thyroxine
tT4: Total Triiodothyronine
TPOAbs: Thyroid Peroxidase Antibodies
TGAb: Thyroglobulin Antibodies
IUGR: Intrauterine Growth Restriction
IUD: Intra Uterine Death
ATA: American Thyroid Association
LBW: Low Birth Weight
